# Data on the auditory duration mismatch negativity for different sound pressure levels and visual perceptual loads

**DOI:** 10.1016/j.dib.2017.02.007

**Published:** 2017-02-08

**Authors:** Stefan Wiens, Malina Szychowska, Rasmus Eklund, Mats E. Nilsson

**Affiliations:** Gösta Ekman Laboratory, Department of Psychology, Stockholm University, Frescati Hagväg 9A, Stockholm, Sweden

**Keywords:** Event-related potential, Mismatch negativity, Perceptual load, Sound pressure level

## Abstract

The data presented in this article are related to our research article entitled “Effects of sound pressure level and visual perceptual load on the auditory mismatch negativity” (M. Szychowska, R. Eklund, M.E. Nilsson, S. Wiens, 2016) [1]. The duration MMN was recorded at three sound pressure levels (SPLs) during two levels of visual perceptual load. In an oddball paradigm (standard=75 ms, deviant=30 ms, within-subjects design), participants were presented with tones at 56, 66, or 76 dB SPL (between-subjects design). At the same time, participants focused on a letter-detection task (find X in a circle of six letters). In separate blocks, perceptual load was either low (the six letters were the same) or high (the six letters differed). In the first data collection, tones had only 76 dB SPL [2]. In a follow-up data collection with exactly the same procedure, tones had 56 and 66 dB SPL [1]. Here, we report the procedure, the recording of electroencephalography (EEG) and its preprocessing in terms of event-related potentials (ERPs), the preprocessing of behavioral data, as well as the grand mean ERPs in figures. For each participant, the reported ERP data include mean amplitudes for standards, deviants, and the difference wave (MMN) at Fz (with tip of nose as a reference), separately for the combinations of SPL and load. Reported behavioral data include the signal-detection measure *d’* as an index of detection performance.

**Specifications Table**

**Table:** 

Subject area	Cognitive Neuroscience
More specific subject area	Mismatch Negativity (MMN)
Type of data	Datasheet (.csv), figures
How data was acquired	Electroencephalography (EEG), event related potentials (ERPs), *d*′ from hit rates and false alarms.
Data format	Preprocessed
Experimental factors	Three SPL groups (56, 66, and 76 dB SPL) – between subjects;
	Two perceptual loads (letter detection task: find X in a circle of 6 letters; low load: all the letters are the same; high load: all the letters are different) – within subjects.
Experimental features	Participants (*N*=83) were presented with a letter detection task that varied in perceptual load (low or high, alternating between blocks). Simultaneously, participants were presented with the tones in an oddball paradigm. For each participant, tones were complex tones with f0=500 Hz presented at 56, 66, or 76 dB SPL (*n*=30, 28, and 25, respectively). Tones differed in duration: standard was 75 ms, deviant was 30 ms. Participants were asked to ignore the tones.
Data source location	Stockholm, Sweden
Data accessibility	Data are provided with this article

**Value of the data**

• Large sample sized study on MMN and perceptual load.
• Useful for meta-analysis on effects of SPL or visual perceptual load on MMN.
• Useful for meta-analysis on effects of auditory distractors on behavioral measures.

## 1 Data

The attached dataset (Supplementary Table 1) includes demographic information for each participants, the dates and times of data collection, mean ERP amplitudes, and behavioral data. The ERP data consist of mean amplitudes for standard, deviant, and their difference (deviant minus standard, i.e., MMN), recorded at Fz with the tip of the nose as a reference, separately for each Sound Pressure Level (SPL) and visual perceptual load. For the behavioral data, we include the signal-detection measure *d*′.

## 2 Experimental design, materials and methods

Participants were 93 students (mean age=25.94, SD=5.83, 52 women) from local universities in Stockholm, Sweden. None of the participants reported hearing problems. Data collection occurred in two waves. The first group of 28 participants was presented with the sounds at 76 dB SPL [2]. Shortly thereafter, we decided to do a follow-up study with lower SPL (66 dB) in order to investigate the effect of SPL on the MMN [1]. At the beginning of this data collection, about 15 participants were presented with tones at 66 dB SPL. Given that many participants expressed interest to participate in our study, we decided to include a second group with even lower SPL (56 dB). After 28 subjects were recruited in this group, we went back to testing mainly participants at 66 dB SPL. Thus, the data collection for the 66-dB and 56-dB groups resembles an ABBA design. This second data collection added 65 participants (at 56 and 66 dB) to the previous 28 subjects (at 76 dB). However, during data processing of all data (from scratch), ten participants were excluded because of excessive ERP artifacts (as explained below). The final sample presented here comprises 83 participants (56 dB, *n*=30; 66 dB, *n*=28; 76 dB, *n*=25).

For both data collections, the procedures, recruitment strategies, and instructions for participants were the same (the only difference was the SPL). Furthermore, the experimental setup (computer, screen, and headphones) was identical. The experiment was programmed in Presentation® Software (Version 14.8, www.neurobs.com). Two experimenters (MS and RE) collected all the data.

The study was approved by the Stockholm section of the Central Ethical Review Board in Sweden and was conducted in accordance with the guidelines in the Helsinki Declaration. All participants gave written informed consent, were debriefed after the experiment, and were compensated with one movie voucher or one subject credit hour.

## 3 Procedure and stimuli

The procedure is identical to that in our previous study [2]. Participants performed a speeded letter detection task (detect X) and were instructed to press the spacebar whenever the letter X was shown (target). Each trial lasted 1 s. On each trial, a ring of six letters was presented for 100 ms. The six letters were drawn randomly without replacement from the set of H, K, M, N, V, W, and Z. The letters were positioned at 2, 4, 6, 8, 10, and 12 o’clock. The size of each letter was 0.53×0.53 degrees (visual angle), the distance between the centers of the letters was 0.98 degrees, and the diameter of the ring (for the centers of the letters) was 3.38 degrees. In the low load condition, all six letters were identical, whereas in the high load condition, all six letters were different. The target letter X appeared on 20% of the trials. On these response trials, six Xs were shown in the low load condition, and one X and five other letters were shown in the high load condition. There were between two and six nonresponse trials before the next response trial.

Participants started with either low or high load (counterbalanced across subjects), then the loads alternated between consecutive blocks. Each block consisted of 250 trials (200 nonresponse trials and 50 response trials). In total, participants performed 1000 nonresponse trials and 250 response trials for each load.

While participants performed the letter detection task, tones were presented simultaneously with the onset of the letter rings. The tones were presented with over-ear headphones (Sennheiser HD 280 Pro). The tones were created in Audacity® [3] software and the SPL was calibrated with Head and Torso Simulator for binaural recordings (Brüel & Kjær (B&K) type 4100) with two internal microphones (B&K type 4190) and pre-amplifiers (B&K type 2669), and ArtemiS Software (Version 12.02.000). The standard tone (75 ms) and the deviant tone (30 ms) were complex tones with f0=500 Hz (higher harmonics at 1000 Hz and 1500 Hz with a drop of 3 dB/harmonic) and 5 ms fade-in and fade-out. Deviant tones occurred on 20% of both response and nonresponse trials. There were between two and six standard tones between two deviant tones.

For each of the 10 blocks for each participant, trial order was determined randomly but with some restrictions. Each block of 250 trials consisted of two sub-blocks of 125 trials each. To obtain 25 deviant trials (i.e., 20%) for these 125 trials, 25 mini-blocks were generated. These mini-blocks consisted of 3, 3, 4, 4, 4, 4, 4, 4, 5, 5, 5, 5, 5, 5, 5, 5, 5, 6, 6, 6, 6, 6, 6, 7, and 7 trials; thus, the sum of the trials=125. The order of the mini-blocks was randomized for every sub-block (of 125 trials) and the first trial of each mini-block was defined as a deviant. That is, if the first three mini-blocks were 6, 3, and 4, then the actual trial order was deviant, five standards, deviant, two standards, deviant, and three standards. Further, for the letter detection task, the requirement was that 20% of the trials should be target trials (i.e., letter X) that required a response (response trials), and this percentage should be similar for deviant and standard trials. Accordingly, for each sub-block of 125 trials, 25 trials should be response trials; of these, 5 should occur during deviants and 20 during standards. For each set of 5 trials, the first, second, or third trial was defined randomly as a response trial. Thus, there were between two and six non-response trials between two response trials. Such trial orders were generated until one of these fulfilled the criterion that only 5 of the response trials occurred during deviants.

Participants were asked to sit as still as possible, to blink as little as possible, and to rest their chin on a chinrest to ensure a constant distance to the computer (57 cm). Participants were instructed to ignore the sounds.

Before the first block, participants practiced the relevant task (low or high load) until they felt that instructions were clear and they were ready to start the main task. At the beginning of each block, between five and eight nonresponse trials with standard tones were presented (these were not counted as actual trials and not analyzed).

## 4 EEG recording

EEG data were recorded from six electrodes at standard 10/20 positions (Fpz, Fz, Cz, M1, M2, and tip of nose) with an Active Two BioSemi system (BioSemi, Amsterdam, Netherlands). Fpz, Fz, and Cz were recorded with pin electrodes in a 64-electrode EEG cap; and M1, M2, and the tip of the nose were recorded with flat electrodes attached with adhesive disks. Two additional, system-specific electrodes were recorded with pin electrodes in the EEG cap: the CMS (between PO3 and POz) served as the internal reference electrode, and DRL (between POz and PO4) as the ground electrode. Data were sampled at 512 Hz and filtered with a hardware low-pass filter at 104 Hz. No high-pass filter was used. All physiological data were processed offline using the FieldTrip toolbox in MATLAB [4]. Continuous data were re-referenced to the tip of the nose.

## 5 Data analysis

For each participant, ERPs were computed for correct rejections (i.e., only trials that did not require a behavioral response and did not evoke a response), separately for each load. Epochs were extracted from 100 ms before tone onset to 400 ms after. Each epoch was baseline corrected with the 100-ms interval before tone onset. For each participant, amplitude ranges (i.e., max minus min) within individual epochs were extracted and visually inspected to exclude apparent outliers. Cutoffs were adjusted individually to retain as many trials as possible while reducing the potential effects of outliers (amplitude ranges did not exceed 120 µV). Inspection was conducted by a researcher who was blind to the condition (stimulus and load) of individual trials, as well as blind to the SPL of each participant. Ten participants were excluded because they had less than 50% remaining trials.

**Fig. 1:**
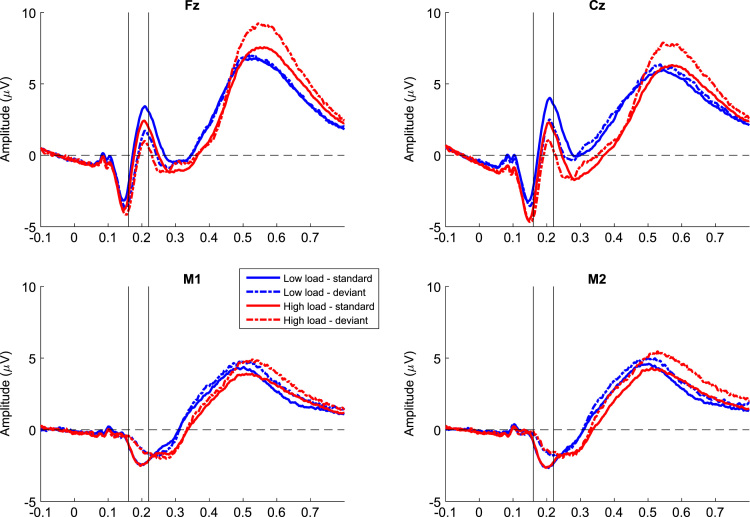
Grand mean ERP waveforms for standards and deviants during low and high load recorded from electrodes at Fz, Cz, M1, and M2. The time of interest for the MMN is between the two vertical lines on each plot.

**Fig. 2:**
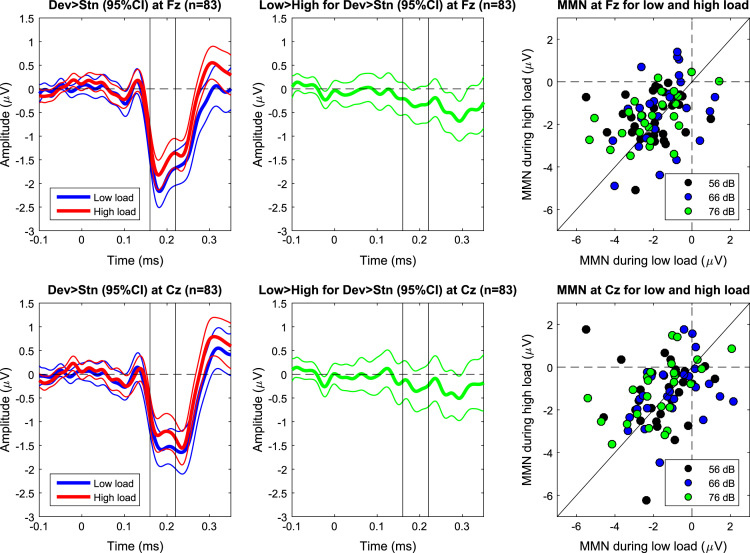
Grand mean waveforms of the MMN (i.e., deviant minus standard) for each load at Fz and Cz (left column), the difference wave in MMN between loads (middle column), and a scatterplot of the mean MMN amplitudes between low and high load for the three SPLs (right column). The time of interest for the MMN is between the two vertical lines in each of the plots in the left and the middle column.

Fig. 1 shows grand mean waveforms of standards and deviants recorded from the different electrodes at Fz, Cz, M1, and M2. To identify the MMN, a difference wave was computed for each participant by subtracting the mean ERP to standards from that to deviants across both load conditions. Across subjects, there was an apparent negativity at the frontal electrodes and a polarity reversal at the mastoids between 160 and 220 ms after tone onset. For this interval (160–220 ms), mean amplitudes were extracted for Fz, Cz, and mastoids for each condition. Fig. 2 shows the grand mean waveforms of the MMN (i.e., deviant minus standard) for each load at Fz and Cz (left column), the difference wave in MMN between loads (middle column), and a scatterplot of the mean MMN amplitudes between low and high load for the three SPLs. For the actual analyses, only the MMN amplitudes at Fz were used because this electrode is used most often [2], [5], and is recommended by guidelines [6].

In the processing of the behavioral data, responses faster than 200 ms were excluded. Because the task had a rapid pace (ITI of 1 s), we were concerned that responses faster than 200 ms may be late responses to the previous trial. Hit rates and false alarm rates were computed for each condition (i.e., tone deviance by load). Signal detection analyses were performed to compute *d*′ [7]. To avoid floor and ceiling effects on hit and false alarm rates, we added 0.5 trial in the numerator and 1 trial in the denominator [8].

Data processing was conducted in Matlab R2015b (The MathWorks, Inc., Natick, Massachusetts, United States.
